# Invisalign technique in the treatment of adults with pre-restorative concerns

**DOI:** 10.1186/2196-1042-14-40

**Published:** 2013-10-20

**Authors:** Gianluca Mampieri, Aldo Giancotti

**Affiliations:** 1 Department of Orthodontics Fatebenefratelli Hospital, University of Rome, Tor Vergata, Rome 00186, Italy

## Abstract

The Invisalign method is gaining an increasing interest as an alternative treatment option in adult patient in multidisciplinary complex cases to simplify the treatment plan. The aim of this work is to show the importance of planning a multidisciplinary approach to respond at the esthetic requests of adult patients and to treat complex cases with high predictability.

## Review

Adult patients seeking orthodontic treatment are increasingly motivated by esthetic considerations. The majority of these patients reject fixed appliances, seeking instead more esthetic treatment options, including lingual orthodontics and thermoformed appliances.

Moreover, adults have often a variety of restorative and periodontal problems that can make them more difficult to treat and sometimes can compromise the results of the treatment. In the majority of these cases, the proper treatment decision should be taken after an overall evaluation on behalf of a 'team’ of an orthodontist, an oral surgeon, a periodontist, and a restorative dentist.

Since the introduction of the Invisalign technique in 1999, only a few clinicians would probably have bet on its rapid success. In fact, the number of patients undergoing orthodontic treatment with clear aligners, both adults and growing patients, has been increasing every year since then.

Following this trend, several clinical papers have been published throughout the last five years, showing the applicability of the technique in correcting various types of malocclusions [1-8].

Despite its growing popularity and its use even in complex cases [7,9,10], questions still remain concerning the proper use of this technique and its limitations. Some of the limitations and disadvantages have been outlined, due to the characteristics of the material and the thermoforming process, which in specific cases can limit or even make the use of clear aligners very difficult.

The aim of this work is to show the use of the Invisalign technique in the treatment of adult patients with restorative concerns and, moreover, the importance of planning an overall approach to match the esthetic requests of the patients and to treat complex cases with high predictability.

### Case 1

#### ***Diagnosis***

A 41-year-old male patient presented with a Class I dental malocclusion and a Class III skeletal pattern. He was particularly concerned about his missing first upper left maxillary molar, and he wished to program an implant replacement.

**Figure 1:**
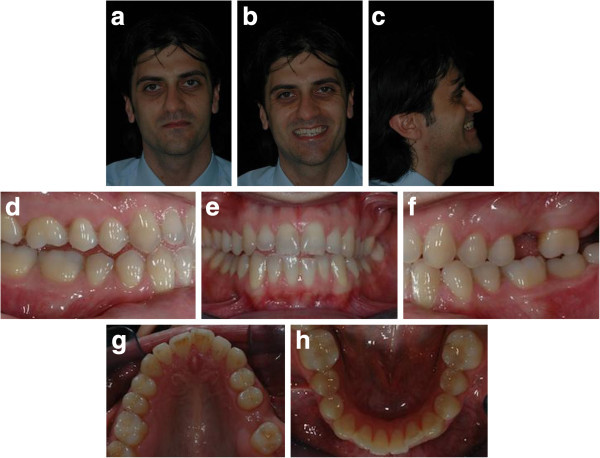
Pre-treatment records (a to h).

**Figure 2:**
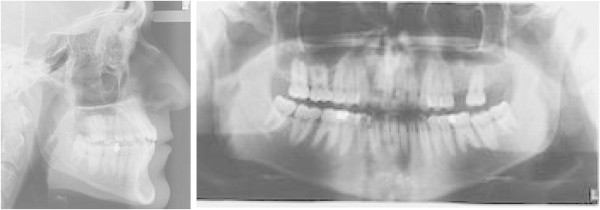
Pre-treatment radiographs.

Intraoral examination showed a light crowding in both upper and lower arches and an anterior and lateral right dental open bite. Both the upper and lower midlines were coincident and centered in the face (Figure 1a,b,c,d,e,f,g,h). The most challenging problems were to gain space on the left side of the upper arch to enable the implant placement of the 2.6 and to correct the dental open bite. Pre-treatment radiographs are evaluated (Figure 2). The patient was seeking a treatment with low esthetic impact.

#### ***Treatment plan***

An accurate examination of the occlusion highlighted a pre-contact of 1.7 as the likely cause of the open bite. The treatment plan concerning the upper arch was the intrusion of 1.7 and the uprighting of 2.7 to gain space for the implant placement. Moreover, the alignment of the upper incisors by means of expansion of the upper arch and pro-inclination of the anterior teeth was planned as well. With regards to the lower arch, the alignment was obtained by interproximal reduction and no pro-inclination of incisors. The extrusion of 1.1, 1.2, and 2.1 was programmed to correct the anterior open bite and to level margins of the upper incisors. In the lower arch, we programmed the sole extrusion of 3.1.

Treatment objectives:

• Intrusion of 1.7 to reduce pre-contact

• Uprighting of 2.7 to gain space for the implant placement

• Expansion of the upper arch and pro-inclination of the anterior teeth

• Extrusion of 1.1, 1.2, and 2.1 to correct anterior open bite and to level margins of the upper incisors

• Extrusion of 3.1

**Figure 3:**
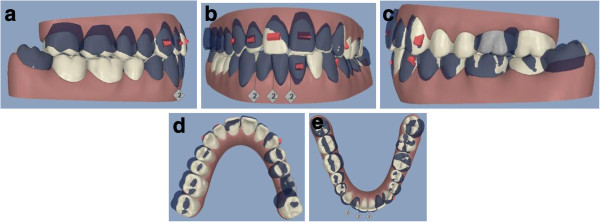
ClinCheck* pre-post superimposition (a to e).

The ClinCheck* projection showed a satisfactory resolution of all occlusal anomalies, with good correction of the open bite and alignment of the anterior teeth (Figure 3a,b,c,d,e).

The first phase of therapy consisted in 15 aligners for the upper arch and 13 for the lower one. During the refinement treatment, the aligners were seven for the upper arch and eight for the lower arch.

#### ***Treatment results***

**Figure 4:**
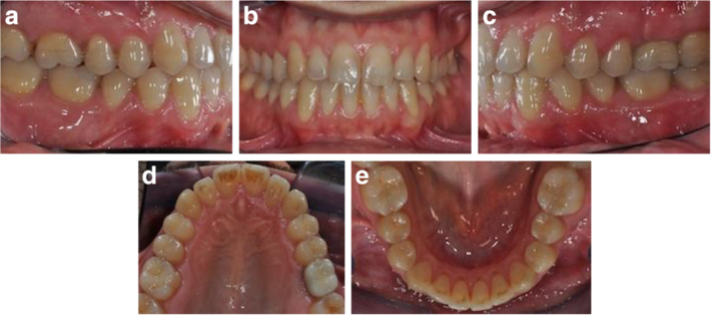
Post-treatment records (a to e).

**Figure 5:**
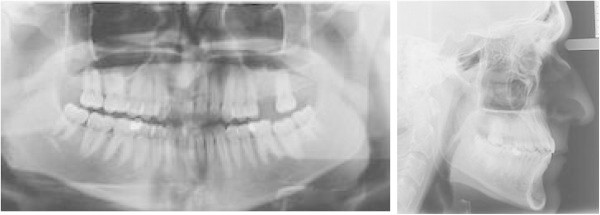
Post-treatment radiographs.

The patient was seen every 4 to 6 weeks (two to four aligners) to check the aligner fit, attachment stability, and cooperation. The final result showed good alignment and occlusion, thanks to the patient’s high cooperation in wearing each aligner for 2 weeks as planned. The upper anterior teeth were aligned; the anterior and lateral open bite was corrected. The second left upper molar was distalized and was obtained space enough for the implant placement of the missing molar (Figure 4a,b,c,d,e). The post-treatment radiographs for final control are requested (Figure 5).

### Case 2

#### ***Diagnosis***

A 37-year-old female patient presented with a dental malocclusion and a Class III skeletal pattern. She wished to improve the esthetic look of her smile, but she did not want a fixed appliance.

**Figure 6:**
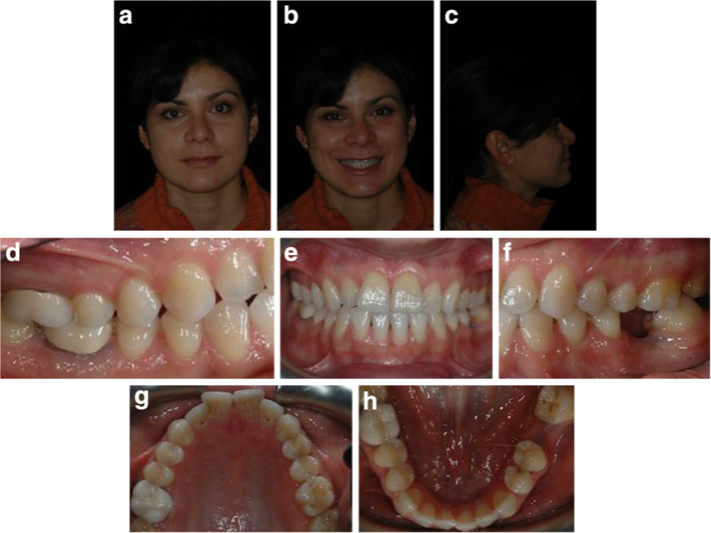
Pre-treatment records (a to h).

**Figure 7:**
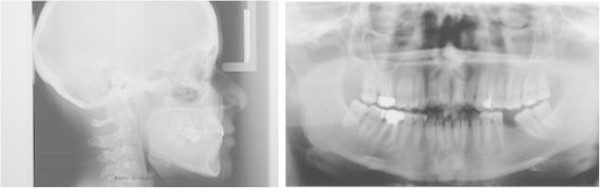
Pre-treatment radiographs.

Intraoral examination showed a light crowding in both arches, Class III dental relationship on the right side and Class I dental relationship on the left side. She presented a missing first lower left molar, and consequently, the second lower left molar was tilted mesially. Both the upper and lower midlines were coincident and centered in the face (Figure 6a,b,c,d,e,f,g,h). Pre-treatment radiographs are evaluated (Figure 7).

#### ***Treatment plan***

The treatment objectives were to resolve the crowding of both arches by expansion of the upper arch, by pro-inclination of the upper incisors and by interproximal reduction (IPR) of the lower incisor. Due to the Class III skeletal pattern, all the mentioned procedures should not have pro-inclined anterior lower teeth.

Further goal included recovering enough space for prosthetic replacement of the missing lower left first molar. This objective would have been achieved by reciprocal movements of the uprighting of the second lower left molar and by mesial movement of the left premolars to level the lower arch. In order to enable the uprighting of the 3.7, the lower left third molar was extracted. The maintenance of 3.8 was considered; however, the uprighting of 3.7 was thought to be faster and more predictable without the third molar.

On the right side, the relationship of Class III was not corrected.

Treatment objectives:

• To resolve the crowding by expansion of the upper arch and by pro-inclination of the upper incisors

• IPR of the lower incisor, not pro-inclined anterior lower teeth

• Uprighting of 3.7 to gain space for the implant placement

• Mesial movement of the left premolars to level the lower arch

**Figure 8:**
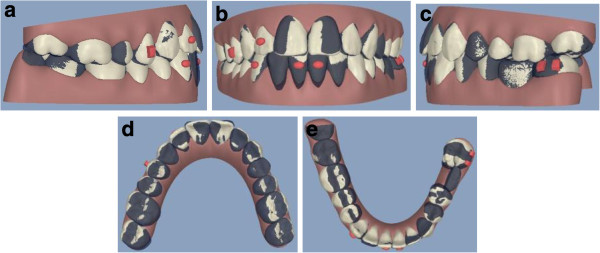
ClinCheck* pre-post superimposition (a to e).

The ClinCheck* projection anticipated satisfactory resolution of all occlusal problems: alignment of both dental arches, uprighting of 3.7, and leveling of the lower arch to gain space enough for implant placement and obtaining a good overjet and overbite (Figure 8a,b,c,d,e).

To ease distal tipping and to provide better control during uprighting of 3.7, two vertical rectangular attachments were bonded on the molar’s buccal side.

The first phase of therapy consisted in 8 aligners for the upper arch and 11 for the lower one. During the refinement phase, the aligners were six for the upper arch and seven for the lower one.

#### ***Treatment results***

**Figure 9:**
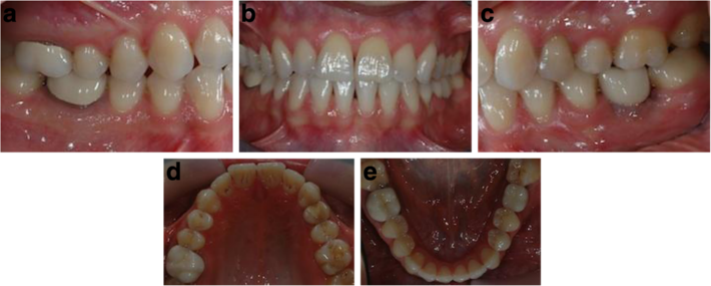
Post-treatment records (a to e).

**Figure 10:**
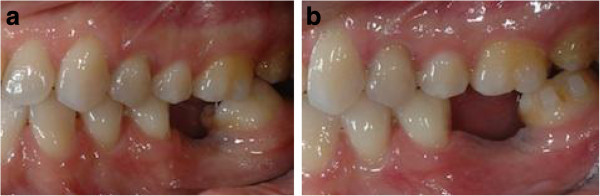
**Molar uprighting (a and b).** The uprighting of 3.7 was obtained, with a good vertical control.

Post-treatment intraoral photographs showed an esthetic and functional improvement, thanks to the patient’s high cooperation in wearing each aligner for 2 weeks as planned. The alignment of both arches was sufficient (Figure 9a,b,c,d,e). The uprighting of 3.7 was obtained, with a good vertical control (Figure 10a,b). Moreover, the use of Invisalign technique enabled to gain space enough for the implant insertion without opening the bite (Figure 9a,b,c,d,e).

The small black spaces between the anterior teeth at the end of orthodontic treatment have been accepted by the patient, considering also the slight exposure at patient smiling.

**Figure 11:**
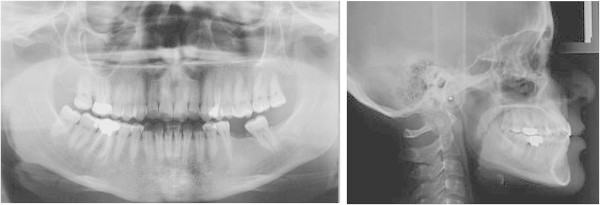
Post-treatment radiographs.

The post-treatment retention has been entrusted to the thermoformed plates Vivera produced by Align*.* The post-treatment radiographs are requested for final control (Figure 11).

### Case 3

#### ***Diagnosis***

**Figure 12:**
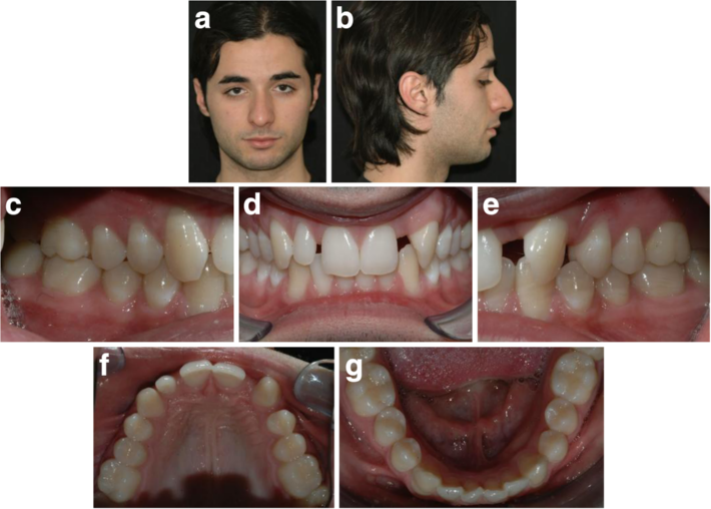
Pre-treatment records (a to g).

**Figure 13:**
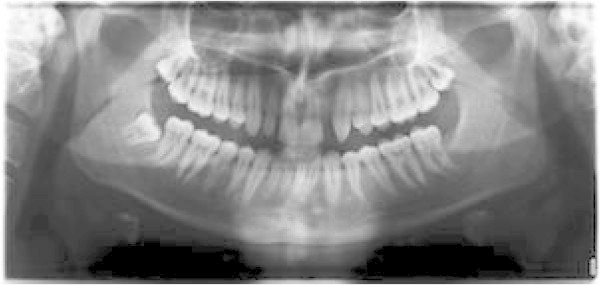
Pre-treatment panoramic X-ray.

A 34-year-old male patient presented with an unpleasant smile. At the intraoral examination, he showed missing left upper lateral incisor, microdontia of right lateral incisor, and upper midline drifted to the left side. In addition, he had a Class I molar and canine on the right side and Class I molar and Class II canine on the left side. He presented a mild deep bite (Figure 12a, b,c,d,e,f,g). Pre-treatment panoramic X-ray is evaluated (Figure 13). The patient was motivated to improve his smile, but he did not want to wear fixed appliance. Therefore, full-arch Invisalign treatment was selected.

#### ***Treatment plan***

The treatment objectives in this case were as follows: primarily, to rearrange anterior upper spaces to ease the solution of the right lateral incisor’s microdontia by esthetic restorative procedures, to open the space for prosthetic replacement of the missing upper left lateral incisor, to center the upper midline with the lower one, and to distalize the left upper canine in order to gain Class I relationship. Further goals included resolving lower crowding and achieving good overjet and overbite.

Treatment objectives:

• To rearrange anterior upper spaces to permit the esthetic restorative of 1.2

• To open the space for prosthetic replacement of the missing 2.2

• To center the upper midline with the lower one

• To distalize 2.3 to gain Class I relationship

• Resolving lower crowding

• Achieving good overjet and overbite

**Figure 14:**
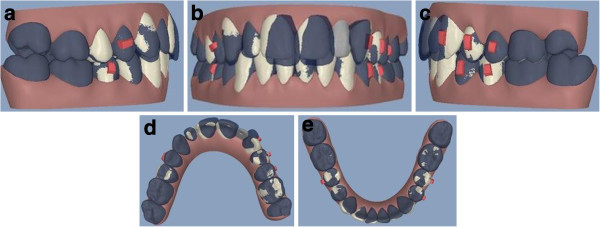
ClinCheck* pre-post superimposition (a to e).

The ClinCheck* projection showed satisfactory resolution of all occlusal anomalies on different levels: achieving enough space for esthetic restoration of the right lateral incisor, properly correcting the upper midline, achieving Class I relationship of 2.3, gaining space in the 2.2 area to enable implant placement, aligning of the lower teeth, and obtaining a good overjet and overbite by intrusion of upper incisors (Figure 14a,b,c,d,e).

The first therapy phase consisted of 23 aligners for the upper arch and 20 for the lower one. During the refinement phase, the aligners were five for both upper and lower arches.

#### ***Treatment results***

**Figure 15:**
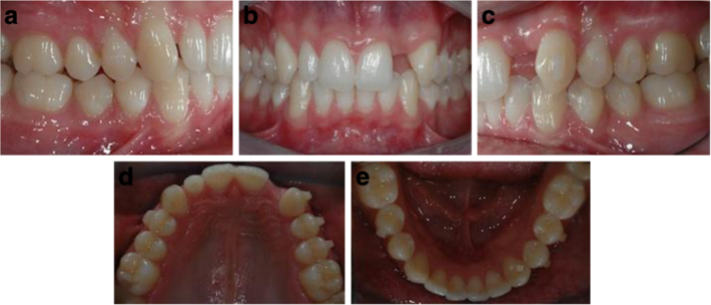
Post orthodontic treatment records before restorative treatment (a to e).

Post-treatment intraoral photographs showed an important and notable esthetic improvement. Correct management of the anterior upper spaces enabled the optimal resolution of the upper lateral incisor’s microdontia by means of restorative procedure. The distalization of the 2.3 led to the achievement of the Class I canine relationship and to the proper space for dental implant placement due to left upper lateral incisor agenesya (Figure 15a,b,c,d,e). The complete space closure distal to the upper left canine would have increased the size of the prosthetic 2.2 with obvious asymmetry of the smile.

**Figure 16:**
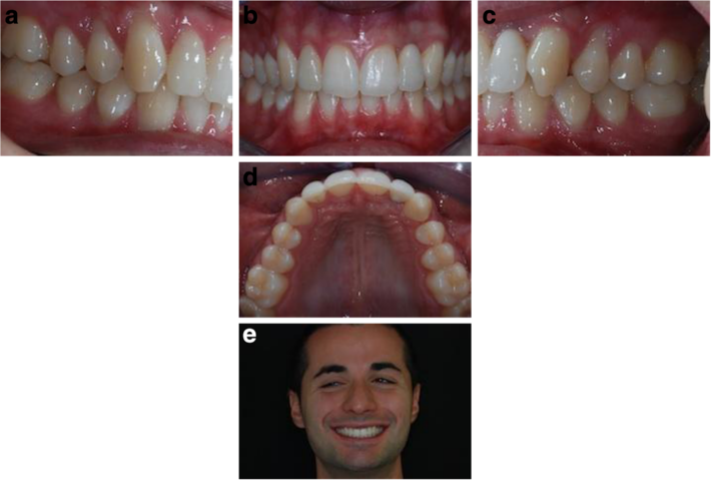
Post-treatment records (a to e).

The upper midline was centered, and overbite was corrected by upper incisors intrusion. Lower crowding was improved by the pro-inclination of the lower incisors. Such movement also eased the overjet and the overbite correction (Figure 16a,b,c,d,e).

**Figure 17:**
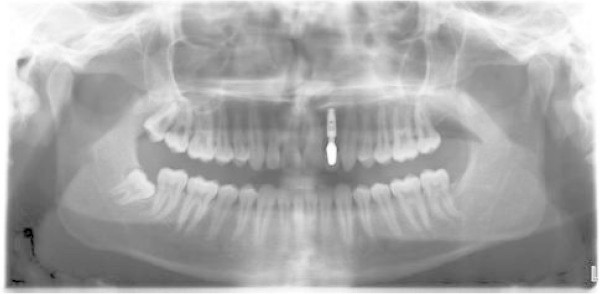
Post-treatment panoramic X-ray.

Comparison of the post-treatment occlusal photographs with ClinCheck* images of their final stage demonstrated the accuracy of the appliance in achieving the desired results (Figure 15a,b,c,d,e). The post-treatment panoramic X-ray is requested for final control (Figure 17).

## Conclusions

As a pre-restorative treatment requires high competence on behalf of different specialists, the orthodontic phase should be performed using truly reliable devices. As illustrated in the following clinical report, the Invisalign technique indeed showed good effectiveness in successfully performing complex adult treatments. All required dental movements were enacted with no relevant counter-effects thanks to high-quality biomechanical features of the aligners. Furthermore, the treatment offered several advantages in terms of maintenance of oral hygiene and comfortable management of the removable appliance. Finally, patient satisfaction was recorded as very high, because they underwent an invisible orthodontic treatment and they reached optimal esthetics and, above all, their occlusion was functionally rehabilitated. In conclusion, for all the abovementioned reasons, we support the use of the Invisalign technique in an increasing number of adult patients with restorative and/or multidisciplinary concerns or needs.

## Consent

Written informed consent was obtained from the patients for the publication of this case report and accompanying images.

## Competing interests

The authors declare that they have no competing interests.

## Authors’ contributions

GM has made the orthodontic treatments and he wrote scientific article. AG has been involved in drafting the manuscript. Both authors read and approved the final manuscript.
